# Price Attractiveness and Price Complexity: Why People Prefer Level-Payment Loans

**DOI:** 10.3389/fpsyg.2021.532696

**Published:** 2021-06-10

**Authors:** Yang Lu, Jian Wang, Chenyang Li, Haoya Huang, Xintian Zhuang

**Affiliations:** School of Business and Administration, Northeastern University, Shenyang, China

**Keywords:** sequence effect, temporal reframing of price, q-exponential discount model, intertemporal choice, discounted utility model

## Abstract

The improving sequence effect suggests that in choices between a rising earning and any other sequences, participants prefer the rising earning. Recent studies show that the improving sequence effect also exists in a loan context. As consumers have a strong preference for falling loan profiles, banks may consider to offer loans in which the loan repayments concentrate at the beginning of the loan term. In this paper, we examined the improving sequence effect in context of a car loan with three repayment plans expressed in temporally reframed prices (TRP). By regressing the evaluation of loan profiles on the perceived price attractiveness, price complexity, TRP and the interaction terms, we find that (1) the perceived price attractiveness and price complexity significantly predict the loan evaluation, and they also explain a significant proportion of variance in loan evaluation; (2) the TRP effect interacts with the improving sequence effect. Specifically, with the introduction of TRP, respondents prefer constant profiles over falling profiles. TRP may explain why level-payment loans are still popular in real world, though the improving sequence effect suggests otherwise.

## Introduction

Firstly introduced by Samuelson (1937), the Discounted Utility Model (hereinafter, DUM) has been widely used to evaluate present utility of future rewards. This theory assumes that individuals evaluate future rewards based on the present value of the rewards by using an exponential discount function. According to the DUM, individuals would prefer falling sequences over rising sequences when evaluating positive future rewards, i.e., individuals prefer rewards received in an decreasing sequence rather than increasing, whilst the total amount of the rewards stays the same. This is because the rewards in a falling sequence concentrate at the beginning of the period, and thus have greater present value than that of a rising sequence of rewards with equal total amount. Similarly, by employing the DUM, we can also conclude that individuals prefer rising sequences over falling sequences if future outcomes are negative.

However, the preference for improvement contradicts the DUM. Loewenstein and Sicherman (1991) first found that when choosing between a falling sequence and a rising sequence of money, whilst the aggregate amount of money of the two sequences was the same, most people preferred the rising sequence. The preference for sequences of monetary rewards has been studied extensively. For positive series of future rewards such as incomes, restaurant visits, leisure activities or other gains, the preference for improvement means that individuals prefer to start with the least attractive outcome and end with the most attractive outcome than the opposite, i.e., they prefer the rising sequence over the falling sequence adding up to the same total amount (Loewenstein and Prelec, 1991, 1993; Loewenstein and Sicherman, 1991; Gigliotti and Sopher, 1997; Thaler, 1999; Matsumoto et al., 2000; Guyse et al., 2002; Duffy and Smith, 2013; Duxbury et al., 2013). Likewise, for negative series of outcomes such as pains, annoying noise, discomfort or other losses, individuals prefer the falling sequence over the rising sequence (Ariely and Loewenstein, 2000; Ariely and Zauberman, 2000; Langer et al., 2005; Rambaud et al., 2018; Garcia et al., 2020).

Some researchers examined human preferences for sequences with respect to loan repayment plans. Hassenzahl (2005) found a preference for decreasing loan profiles. Participants were requested to take out a loan for a vacation, and to choose between a profile starting with a large repayment followed by a series of small repayments, and a profile ending with the large repayment. The majority of respondents preferred an earlier large repayment. Hoelzl et al. (2011) viewed loan repayments as a sequence of installments that are either falling, rising or constant over time. The respondents preferred the falling repayment plan over other options, and they took out loans that contradicted their financial benefits. Rambaud et al. (2019) also found a strong preference for falling sequence in car loans, and used the q-exponential discounting to explain the improving sequence effect.

In real world, marketers continually tried to minimize the perceived cost of a product. A common practice is the temporal reframing of prices (hereinafter, TRP), in which the price is expressed by marketers according to a short period, such as car insurance for “$1 a day” as opposed to “$365 a year,” despite of the fact that the physical cash flows of the payments remain the same. In an initial study, Gourville (1998) referred to this technique as “pennies-a-day.” Gourville (1998, 1999) found that consumers’ purchase intentions increased in domains such as charitable donations, cellular telephone services, and health clubs memberships, when the prices were expressed in a per-day form. Gourville (2003) examined the reframed prices of three periods, and found that both per-day and per-month forms were preferred to a per-year form. Bambauer-Sachse and Grewal (2011) examined the role of four moderating variables, and found that per-day reframed prices were more beneficial than aggregate prices for high-priced products, especially in combination with even price endings, a comparatively short time period, or customers with poor calculation affinity.

However, Bambauer-Sachse and Mangold (2009) showed the negative effects of TRP on product evaluations. They found that TRP has positive effects through higher price attractiveness but negative effects through higher complexity of the price structure and a stronger feeling of being manipulated by the marketer. Specifically, price attractiveness positively influences loan evaluations. Previous studies show that objective price presentation influences price perceptions, which affect perceived product quality, value, and willingness to buy (e.g., Dodds et al., 1991; Grewal et al., 1998; Gourville, 2003). If TRP has a positive effect on perceived price attractiveness, it then should result in better evaluations and purchase intentions. In contrast, price complexity negatively influences loan evaluations. According to equity theory (Adams, 1965; Martins and Monroe, 1994), the greater complexity of the temporally reframed price structure implies that more cognitive input is needed, relative to the output gained from the product. Thus, more complex price structures may cause consumers to suspect they are being manipulated by marketers, prompting comparatively negative product evaluations. Price complexity therefore captures both the complexity of price structure and a feeling of being misled (Bambauer-Sachse and Mangold, 2009; Bambauer-Sachse and Grewal, 2011).

The main objective of this paper is to examine the improving sequence effect in a loan context by employing TRP technique. The repayment plans of the loan are expressed in per-day forms and per-year forms. We use perceived price attractiveness to represent the positive effect of TRP, and perceived price complexity to represent the negative effect of TRP. As Bambauer-Sachse and Grewal (2011) stated, per-day reframed loan profiles are perceived as more attractive relative to per-year reframed loan profiles, and thus may result in better evaluation due to this positive effect of TRP. However, they are also perceived as more complex at the same time, and may as well be less preferred due to the negative effect of TRP. The overall evaluation of a loan profile depends on the joint role of price attractiveness and price complexity.

Temporally reframed prices may also interact with the improving sequence effect. According to the improving sequence effect, individuals prefer falling over rising and constant loan profiles. However, some research also detected a strong preference for constant sequences (e.g., Read and Powell, 2002; Hoelzl et al., 2011). Read and Powell (2002) related the preference for constant sequences to “the ease with which money can be managed.” This explanation is closely related to price complexity in TRP. A logical deduction is that if the constant loan profile is considered as an easier way to manage money, it may also be perceived as less complex than other profiles. Particularly, marketers can express constant loan profiles using a per-day loan cost, but they have to use a series of falling or rising per-day costs when describing falling or rising profiles. A series of prices are usually considered as more complex than a single price, and then constant profiles will be preferred due to less price complexity. Thus, we hypothesize that the effect of TRP differs across profiles. Specifically, the introduction of the per-day framings affects price complexity of constant loan profiles differently than other profiles. This may explain the popularity of level-payment loans in real-life banking service, as they benefit from less price complexity. Hence, the main objective of this study is to explore the interaction effect between the improving sequence effect and the TRP effect. The foregoing discussion generates the following testable hypotheses:

H1. Ratings of loan profiles are positively correlated with perceived price attractiveness, and negatively correlated with perceived price complexity.
H2. Per-day reframed loan profiles are perceived to be more attractive than per-year reframed profiles.
H3. Per-day reframed loan profiles are perceived to be more complex than per-year reframed profiles for falling and rising profiles, but not for constant profiles.
H4. An interaction effect exists between the improving sequence effect and the TRP effect. When loan profiles are expressed in a per-day form, individuals prefer constant loan profiles over falling and rising loan profiles.

The organization of this paper is as follows. In Section “Methodology,” we explain the empirical methodology. In Section “Results,” we regress scores of loan profiles on price attractiveness, price complexity, TRP and the interaction terms. We present our conclusions in Section “Discussion.”

## Methodology

### Material

We conducted this experiment in the same way as Hoelzl et al. (2011) and Rambaud et al. (2019). Participants read scenarios which described that they worked for a big company and earned 10,000 Yuan per month after taxes (1USD≈7 Yuan or $1≈¥7, ¥10,000≈$1,400). They will stay in this job for at least three years. They were asked to consider purchasing a new car that costs ¥120,000 (≈$17,000) on credit. Research shows that per-day framings are more beneficial for products consumed on an ongoing basis than on a lump sum basis (Gourville, 1999), and for high-priced products than low-priced products (Bambauer-Sachse and Grewal, 2011). As cars are expensive and consumed on a continuous basis, we expected that the respondents would prefer the per-day reframed car loans. The loan value was the same as the price of the car with three optional repayment plans (i.e., constant installments, falling installments or rising installments), and with regard to two annual interest rates (10 vs. 0%). The loan is three-year term. Both Hoelzl et al. (2011) and Rambaud et al. (2019) used 5-year loan term in their experiments, but 3-year term is more common in China’s auto loan market. The loan was repaid in monthly installments. The monthly principal repayments of the falling plan were ¥5,000 (¥60,000/12) in year 1, ¥3,333.3 (¥40,000/12) in year 2, and ¥1,666.7 (¥20,000/12) in year 3. The monthly principal repayments of the rising plan were ¥1,666.7 in year 1, ¥3,333.3 in year 2, and ¥5,000 in year 3. We adopted similar amortization schedule as Rambaud et al. (2019) except for constant profiles. Both Hoelzl et al. (2011) and Rambaud et al. (2019) designed the constant profiles by fixing the monthly principal repayment. The monthly payments of such constant profiles are actually a falling sequence, as the monthly payment of interest falls over time. In contrast to these studies, our experiment defined the constant sequence as a level payment loan with identical monthly payments (principal + interest) over the term of the loan [see equation (1)].

(1) $${\text{MP}}_{\text{c}}=\text{L}\,\left(\frac{r_{\text{L}}\,{\left(1+r_{\text{L}}\right)}^{t}}{{\left(1+r_{\text{L}}\right)}^{t}-1}\right)$$

where *MP*_*c*_ is the constant monthly payment, *L* is the loan principal, *r*_*L*_ is the loan rate, *t* is the number of installments of this loan, *t*∈[1,2,…,n].

The loan profiles were presented with per-year repayments or per-day repayments. Although repayments are temporally reframed, the respondents still pay off the loan on a monthly basis. A per-year reframed repayment is the sum of the twelve actual monthly payments in that year, and the per-day reframed repayment is the per-year reframed repayment/365 (see Supplementary Appendix A).

### Participants

144 MBA students (76 males and 68 females) from Northeastern University (China) with a mean age of 29.48 years took part in the experiment.

### Measures

All items were measured on a seven-point rating scale from 1 to 7. At first, participants were asked to evaluate each loan plan, where “1” was the score for a loan they would never choose and “7” was the score for what they considered to be the best plan. Next, they were required to respond to two questions regarding the profiles: price attractiveness (“not at all attractive/extremely attractive”), and price complexity (“not at all complex/extremely complex”). These scales were derived from previous studies (e.g., Bambauer-Sachse and Grewal, 2011; Bornemann and Homburg, 2011; Hoelzl et al., 2011; Shirai, 2018; Rambaud et al., 2019).

### Procedure

The questionnaires (see Supplementary Appendix B) were presented in a paper-pencil-version at Northeastern University (China), and were distributed in MBA classes. Participants were asked to assign scores to the three repayment plans at two interest rates and at per-day or per-year framings. They were randomly assigned to one of the four experimental groups via the questionnaires (2 rates × 2 temporal framings), which were also randomized. We decided the sample size according to the number of MBA students. Also, we designed our study to let each group have the same number (36) of participants for comparison’s sake, thereby resulting in an analytic sample of 144 (36 × 4) participants.

Participants were allowed to assign the same score to the three plans. They were then requested to evaluate price attractiveness and price complexity of the profiles using a 1 to 7 scale. At the beginning of the experiment, the researcher explained the procedure. The experiment took approximately 15 min to complete. No monetary incentive was given for participation.

Finally, to offset the impact of stylized responses, the order of presentation of the profiles was counterbalanced across subjects. Therefore, for those 36 subjects in each group, 12 saw falling, constant and rising profile from left to right, 12 saw constant, rising, and falling profile from left to right, while 12 saw rising, falling and constant profile from left to right.

## Results

### Interaction Effect Between the Improving Effect and TRP Effect

#### Means of Evaluations

Participants evaluated the rising profile as the least preferred option regardless of the loan rate and temporal framings. This result provides additional support for the improving effect (Loewenstein and Prelec, 1993), and is consistent with the result of Hoelzl’s (2011) study. The preference order of per-year reframed profiles at 10% discount rate (falling > constant > rising) was consistent with the order deduced from utilizing the DUM and exponential discounting. However, the preference orders of the other three groups contradicted the DUM. Table 1 shows the group means of scores, the perceived price complexity and price attractiveness of the profiles.

**TABLE 1:** Means of evaluations.

**Groups**	**Number of subjects**	**Sequence Profiles**								
		**Falling**			**Constant**			**Rising**		
		**Score**	**PC**	**PA**	**Score**	**PC**	**PA**	**Score**	**PC**	**PA**
Per-year, 0%	36	5.19 (1.62)	1.69 (1.43)	4.08 (1.68)	5.64 (1.10)	1.67 (1.76)	4.94 (1.37)	3.81 (1.6181)	1.61 (1.34)	3.14 (1.85)
Per-day, 0%	36	3.86 (1.74)	3.19 (1.97)	4.97 (1.25)	5.75 (1.32)	1.72 (1.45)	5.58 (1.32)	3.14 (1.81)	3.33 (2.11)	4.11 (1.47)
Per-year, 10%	36	5.19 (1.95)	1.56 (0.88)	4.56 (1.65)	4.92 (1.44)	1.64 (1.38)	4.69 (1.77)	3.50 (1.63)	1.81 (1.53)	2.39 (1.73)
Per-day, 10%	36	4.64 (1.62)	2.97 (1.08)	4.86 (1.46)	5.28 (1.26)	1.61 (0.77)	5.25 (1.23)	2.56 (2.08)	2.75 (1.52)	2.92 (2.26)

*Score is the overall evaluation of loan profiles, PC is the perceived price complexity, and PA is the perceived price attractiveness (with standard deviations in parentheses).*

#### ANOVA Results

**FIGURE 1:**
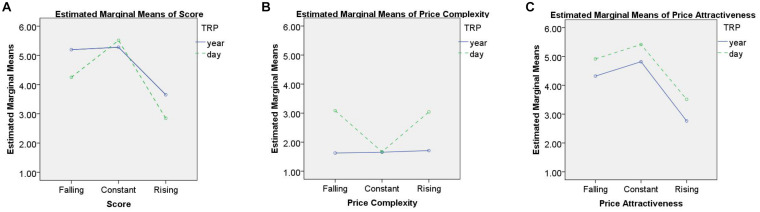
Estimated marginal means (day vs. year). **(A)** Score, **(B)** price complexity, and **(C)** price attractiveness.

We analyzed the means using 3(sequences) × 2(TRP) × 2(Interest) ANOVAs. Normality is not an issue for our large sample size. According to central limit theorem, for sufficiently large samples with size greater than 30 (144 in our study), the sampling distribution for means is always normally distributed regardless of a variable’s original distribution. Because the loan profiles have roughly equal standard deviations, ranging from 1.3 to 1.9, the assumption of homoscedasticity is also met. We run the tests in SPSS version 20. The sequence score, perceived price complexity, and perceived price attractiveness were used as the dependent variables (a within-subject factor). The independent variables included the interest rate (10%, or 0%), and TRP (day-framing or year-framing), which are all between-subjects factors. A Tables 2–4 show the results of the ANOVAs. Figures 1A–C show the estimated marginal means.

**TABLE 2:** ANOVA results for evaluation score.

**Factor**	**DF1**	**DF2**	**F**	**MS_between_**	**MS_within_**	**η_*p*_^2^**
Sequence	2	264	54.936***	173.419	3.157	0.282
TRP	1	140	17.433***	27.502	1.578	0.111
Interest	1	140	3.241	5.113	1.578	0.023
Sequence × TRP	2	264	4.748**	14.988	3.157	0.033
Sequence × Interest	2	264	3.213*	10.141	3.157	0.022
TRP × Interest	1	140	1.070	1.688	1.578	0.008
Sequence × TRP × Interest	2	264	0.794	2.507	3.157	0.006

Columns list the degrees of freedom for the numeration (DF1), and denominator (DF2), the F ratio (F), the mean-squared between (MS_*between*_), the mean-squared within (MS_*within*_), and the partial eta squared (η_*p*_^2^). *p < 0.05, **p < 0.01, ***p < 0.001.

**TABLE 3:** ANOVA results for price complexity.

**Factor**	**DF1**	**DF2**	**F**	**MS_between_**	**MS_within_**	**η_*p*_^2^**
Sequence	2	264	19.910***	23.863	1.199	0.125
TRP	1	140	22.467***	94.454	4.204	0.138
Interest	1	140	0.564	2.370	4.204	0.004
Sequence × TRP	2	264	19.238***	23.058	1.199	0.121
Sequence × Interest	2	264	0.141	0.169	1.199	0.001
TRP × Interest	1	140	0.637	2.676	4.204	0.005
Sequence × TRP × Interest	2	264	1.207	1.447	1.199	0.009

Columns list the degrees of freedom for the numeration (DF1), and denominator (DF2), the F ratio (F), the mean-squared between (MS_*between*_), the mean-squared within (MS_*within*_), and the partial eta squared (η_*p*_^2^). ***p < 0.001.

**TABLE 4:** ANOVA results for price attractiveness.

**Factor**	**DF1**	**DF2**	**F**	**MS_between_**	**MS_within_**	**η_*p*_^2^**
Sequence	2	264	58.420***	152.521	2.611	0.294
TRP	1	140	17.639***	45.370	2.572	0.112
Interest	1	140	5.475*	14.083	2.572	0.038
Sequence × TRP	2	264	0.107	0.280	2.611	0.001
Sequence × Interest	2	264	4.631*	12.090	2.611	0.032
TRP × Interest	1	140	1.440	3.704	2.572	0.010
Sequence × TRP × Interest	2	264	0.230	0.600	2.611	0.002

Columns list the degrees of freedom for the numeration (DF1), and denominator (DF2), the F ratio (F), the mean-squared between (MS_*between*_), the mean-squared within (MS_*within*_), and the partial eta squared (η_*p*_^2^). *p < 0.05 and ***p < 0.001.

Figure 1 shows the estimated marginal means of falling, constant and rising profiles with regard to per-day and per-year framings. The lines in Figures 1A,B are far from parallel, suggesting an interaction effect between the improving sequence effect and the TRP effect, i.e., the improving sequence effect is different for per-year reframed and per-day reframed profiles.

Table 2 shows that the main effects of Sequence and TRP are significant, suggesting the existence of the improving sequence effect and TRP effect. The results also show a significant Sequence × TRP interaction, and a significant Sequence × Interest interaction. To identify the locus of Sequence × TRP interaction, we examined the effect of sequence for per-day and per-year framings separately. At per-year framings, *F* = 21.257, *p* < 0.001, η_*p*_^2^ = 0.23. Pair comparisons show that the rising profile differs from the falling profile (mean difference = −1.542, *p* < 0.001) and the constant profile (mean difference = −1.625, *p* < 0.001). The difference between the falling and constant profile, however, is not statistically significant (mean difference = −0.08, *p* > 0.05). In contrast, at per-day framings, *F* = 35.923, *p* < 0.001, η_*p*_^2^ = 0.336. All three profiles are significantly different from each other. The rising profile differs from the falling profile (mean difference = −1.403, *p* < 0.001) and the constant profile (mean difference = −2.667, *p* < 0.001). The falling profile differs from the constant profile (mean difference = −1.264, *p* < 0.001). In general, the sequence effects are significant regardless of TRP involved in the profile. Tables 1–4 show that the rising profile is with the least score in all conditions, indicating that participants are not financially rational and the DUM is violated. This result provides additional support for the improving sequence effect (Loewenstein and Prelec, 1993), and is consistent with the results of Hoelzl’s (2011) study and Rambaud et al.’s (2019) study. However, individuals prefer the constant profile over the falling profile at per-day framings.

Table 3 shows a significant main effect of TRP, indicating that a per-day reframed price is generally perceived to be more complex than a per-year reframed price for falling and rising profiles. Thus, H3 is supported. There is also a significant interaction effect between Sequence and TRP for price complexity. We examined the sequence effect for per-day and per-year framings separately. At per-year framings, *F* = 0.138, *p* > 0.05, η_*p*_^2^ = 0.002. Pair comparisons suggest that individuals perceive all three profiles as equally complex. Neither the difference between the rising and falling profile (mean difference = 0.083, *p* > 0.05), the difference between the rising and constant profile (mean difference = 0.056, *p* > 0.05), nor the difference between the falling and constant profile (mean difference = −0.028, *p* > 0.05) is significant. In contrast, At per-day framings, *F* = 32.399, *p* < 0.001, η_*p*_^2^ = 0.316. Pair comparisons show that the constant profile differs from the falling profile (mean difference = −1.417, *p* < 0.001) and the rising profile (mean difference = −1.375, *p* < 0.001). But the difference between the falling and rising profile is not significant (mean difference = 0.042, *p* > 0.05). The result indicates that the constant profile is perceived to be less complex only when the loan profiles are expressed in a per-day form. This result is consistent with the result of Table 2, as the falling profile is preferred when the profiles are described in a per-year form.

Table 4 shows that using a per-day reframed price leads to a significantly more positive perception of price attractiveness than using a per-year reframed price, as the main effect of TRP is also significant. Therefore, H2 is supported. No significant interaction effect between Sequence and TRP is found.

As Tables 2–4 show significant sequence x TRP interactions in score and price complexity, we examined the main effect of TRP for each sequence. Table 5 shows that TRP affects score, price attractiveness, price complexity of falling and rising loan profiles. However, TRP does not significantly affect score and price complexity of constant profiles. This finding is consistent with the mean values in Table 1, in which the mean score of constant profiles in a per-day form is not significantly different from that in a per-year form. However, the mean score of constant profiles is significantly higher than the mean scores of falling and rising profiles when all profiles are described in a per-day form. A possible explanation is that constant profiles are positively affected by TRP in terms of higher price attractiveness just like falling and rising profiles. But unlike other profiles, when switching from a per-year form to a per-day form, constant profiles are not perceived to be more complex, i.e., the falling and rising profiles are exposed to both positive and negative effects of TRP, while the constant profile only benefits from the positive effect of TRP. Therefore, H4 is supported.

**TABLE 5:** TRP effect for each sequence.

**Evaluations**	**DF1**	**DF2**	**F**
Scores of falling profiles	1	142	10.488***
Scores of constant profiles	1	142	1.166
Scores of rising profiles	1	142	7.249**
PA of falling profiles	1	142	5.573*
PA of constant profiles	1	142	6.241*
PA of rising profiles	1	142	5.584*
PC of falling profiles	1	142	39.337***
PC of constant profiles	1	142	0.004
PC of rising profiles	1	142	23.438***

*p < 0.05, **p < 0.01, ***p < 0.001.

### Regression Analysis Between Scores of Loan Profiles, Price Attractiveness and Price Complexity

As the main focus of this study is to explore the interaction between the improving sequence effect and TRP effect, we treated TRP as a between-subjects factor in our experiment and ran hierarchical multiple regression analysis with one dependent variable (scores). In model 1, two independent variables were included: price complexity and price attractiveness. Table 6 shows the results of regression tests (we run the tests in SPSS version 20.). Coefficients of price attractiveness are positive and coefficients of price complexity are negative for all loan profiles. All coefficients are statistically significant except for that of price complexity for constant profiles. The exception is possibly because the per-day and per-year framings have close mean price complexities (see Table 1). These variables accounted for a significant amount of variance in scores. For falling profiles, *R*^2^ = 0.421, *F*(2, 141) = 51.246, *p* < 0.001; for constant profiles, *R*^2^ = 0.413, *F*(2, 141) = 49.578, *p* < 0.001; for rising profiles, *R*^2^ = 0.417, *F*(2, 141) = 50.378, *p* < 0.001.

**TABLE 6:** Regression results.

	**Falling**		**Constant**		**Rising**	
	**Coeff.**	**VIF**	**Coeff.**	**VIF**	**Coeff.**	**VIF**
**Model 1**						
Attractiveness	0.687 (0.075)***	1.004	0.571 (0.058)***	1.005	0.602 (0.062)***	1.028
Complexity	−0.364 (0.074)***	1.004	−0.062 (0.062)	1.005	−0.271 (0.067)***	1.028
*F*(2,141)	51.246***		49.578***		50.387***	
R^2^	0.421		0.413		0.417	
**Model 2**						
Attractiveness	0.677 (0.084)***	1.236	0.572 (0.058)***	1.005	0.640 (0.060)***	1.059
Complexity	−0.361 (0.075)***	1.029	−0.057 (0.062)	1.013	−0.337 (0.067)***	1.103
Attractiveness * Complexity	−0.017 (0.062)	1.248	0.034 (0.043)	1.009	0.139 (0.037)***	1.090
*F*(3,140)	33.967***		33.182***		41.505***	
ΔF	0.079		0.641		14.262***	
R^2^	0.421		0.416		0.471	
ΔR^2^	0.000		0.003		0.054	
**Model 3**						
Attractiveness	0.676 (0.114)***	2.543	0.489 (0.092)***	2.730	0.864 (0.089)***	2.769
Complexity	−0.210 (0.134)***	3.668	0.072 (0.077)	1.676	−0.481 (0.111)***	3.574
Attractiveness * Complexity	−0.017 (0.062)	1.405	−0.026 (0.050)	1.472	0.123 (0.036)***	1.211
TRP	−1.079 (0.255)***	1.356	−0.088 (0.172)	1.124	−0.884 (0.233)***	1.262
Interest	0.212 (0.222)	1.023	−0.457 (0.165)**	1.027	0.404 (0.222)	1.147
Complexity × TRP	0.028 (0.172)	3.378	−0.423 (0.154)**	2.346	0.352 (0.135)**	3.037
Attractiveness × Interest	0.110 (0.146)	2.097	0.031 (0.117)	2.411	−0.250 (0.115)*	2.525
*F*(7,136)	19.120***		17.615***		25.006***	
ΔF	4.052**		3.249**		9.079***	
R^2^	0.496		0.476		0.563	
ΔR^2^	0.075		0.063		0.146	

*p < 0.05, **p < 0.01, ***p < 0.001. Coeff. is unstandardized regression coefficient (standard error).

In model 2, we centered price complexity and price attractiveness, and used the multiply as the third independent variable to examine the moderation. The interaction term between price complexity and price attractiveness was added to the regression model. For rising profiles, the interaction term is significant, and model 2 accounts for significantly more variance than model 1, ΔR^2^ = 0.054, ΔF = 14.262, *p* < 0.001. This result shows that the effect of price attractiveness is higher when the perceived price complexity is high, relative to the effect when the perceived price complexity is low. However, the interaction term is not significant for falling or constant profiles.

In model 3, we included TRP and interest rate as independent variables, TRP = 0 for per-year reframings, TRP = 1 for per-day reframings, Interest = 0 for 0%, Interest = 1 for 10%. Complexity × TRP and Attractiveness × Interest interactions were also included because of the significant interaction effects (see Tables 2–4). Model 3 accounts for significantly more variance than model 1 for all profiles (*p* < 0.01 for falling and constant profiles, *p* < 0.001 for rising profiles). Although price attractiveness and price complexity captures most of the changes in scores, TRP and interest rate also influence evaluations of loan profiles.

In general, scores are positively correlated with the perceived price attractiveness, and negatively correlated with the perceived price complexity. The inclusion of covariates such as TRP and interest rate significantly increase the R^2^, but price attractiveness and price complexity account for most of the variance in scores in all three models. No multicollinearity was detected. Therefore, H1 is supported.

## Discussion

In this study, we examined the preferences for sequences in context of a car loan when the loan repayment plans are expressed in temporally reframed prices. Our study is motivated by the fact that TRP tactic has been widely used as an effective pricing strategy to improve consumer’s product evaluations. In general, our results show that TRP has positive effects through higher price attractiveness but negative effects through higher price complexity. The results also support the improving sequence effect. Also, we found an interaction effect between the improving sequence effect and TRP. Although TRP tactic improves price attractiveness for all loan profiles, it affects price complexity differently. Specifically, the introduction of TRP leads to higher price complexity for falling and rising loan profiles, but has no significant influence on constant profiles. Thus, individuals choosing among loan repayment profiles expressed in per-day forms will prefer constant profiles.

A number of research papers provided explanations for preferences in relation to money sequences (e.g., Loewenstein and Sicherman, 1991; Chapman, 1996, 2000; Read and Powell, 2002). Many studies believe that the violation of the DUM is caused by the misuse of exponential discount function. They explained the improving sequence effect by employing discount functions other than exponential discounting. For example, hyperbolic discounting (Loewenstein and Prelec, 1993; Overton and MacFadyen, 1998) and the q-exponential discounting (Rambaud et al., 2019) were used. Rambaud et al. (2019) stated that the falling profile is more appealing if participants discount future loan repayments using the q-exponential discounting instead of the traditional exponential function. The q-exponential discount function is known in the deformed algebra inspired in non-extensive thermodynamics (Tsallis, 1994), and was first utilized to study intertemporal choices, as proposed by Cajueiro (2006).

(2) $$V\left(L\right)=\sum_{t=1}^{n}{\text{MP}}_{\text{t}}/\left[1+\left(1-\text{q}\right){\text{r}}_{\text{q}}\text{t}\right)]{}^{1/\left(1-\text{q}\right)}$$

where *t*, *L* stay the same, *MP*_*t*_ is the monthly repayment, *V*(*L*) is subject discounted value of the repayments, and *r*_*q*_ and *q* are discount parameters of the model, *t*∈[1,2,…,n]. For *q*→1, the q-exponential discount recovers the classical exponential discount. For *q*→0, it yields the simple hyperbolic discount (Cajueiro, 2006). Hence, with two free parameters, the q-exponential discount model is a general form of the exponential discount model and simple hyperbolic model, in which 1-*q* indicates the degree of inconsistency (Takahashi et al., 2007). If 1-*q* > 0, q-exponential discounting exhibits decreasing impatience, “the instantaneous discount rate is decreasing according to the value of *q*” (Rambaud and Torrecillas, 2013). Because the discount factor of the q-exponential discount function between adjacent periods is smaller than between similar periods that are further away, the discount rate of the q-exponential discount function is higher than that of the exponential discount function at the beginning of the loan term, but is lower in the long run.

The inconsistency level can be calculated as the coefficient of variation (CV) of the obtained average scores (see Table 1): 1-*q* (CV) for the four groups (Per-year, 0%, Per-day, 0%, Per-year, 10%, Per-day, 10%) are 0.1954, 0.3172, 0.2001, and 0.3419, and all greater than 0. Due to this time inconsistency, the falling profile is more appealing if participants discount *MP*_*t*_ using the q-exponential function instead of the exponential function, as the former function results in a small present value. This type of thinking was labeled as “optimization” by Read and Powell (2002), because individuals can always maximize their utilities by choosing the sequence with the highest present value of positive outcomes (Samuelson, 1937), or lowest present value in context of a loan.

However, some empirical results contradicts the “optimization” theory. For example, studies also found the improving sequence effect in the context of interest-free loans (Hirst et al., 1992; Wonder et al., 2008; Hoelzl et al., 2011). As Rambaud et al. (2019) also stated, no discount function can explain the improving sequence effect if the interest rate is zero. As the rising profile will always has the least subjective discounted value regardless of discount function, it should represent respondents’ best choice. Moreover, individuals may have limited financial capability to discount future outcomes. Herrmann and Wricke (1998) found that when evaluating the attractiveness of auto loan offers, respondents did not even calculate the product of monthly payment and number of payments, not to mention using discounted values. “Optimization” cannot explain the preference pattern in our result either, as the introduction of TRP does not change the physical cash flows of the payments, the discounted values of the per-day and per-year reframed loan profiles are identical regardless of discount function.

A possible explanation is that consumers do not process price information completely but use simplifying heuristics (Anderson, 1971; Davis et al., 1986; Bambauer-Sachse and Mangold, 2009). Therefore, they may evaluate loan profiles on the basis of the reframed price and predict a lower total cost. Furthermore, consumers may compare the per-day loan cost to the cost of a petty cash expense. For example, an advertisement for smart phones stated “For the Cost of Your Morning Coffee, Never Be Un-Reachable!.” Likewise, a per-day reframed constant loan profile can also be compared to a breakfast or a pack of cigarettes. TRP induces consumers to compare the per-day loan cost to a petty cash expense or daily budget, and thus influences their perceptions of product affordability. For example, the per-day expressed constant profile at 0% loan rate in our study is only ¥109.6 (≈$15) per day, very close to the expense of a good lunch or a pack of top brand cigarette, easily fitting into many respondents’ daily budgets. Gourville (1999)’s result shows that an explicit petty cash comparison (e.g., one’s morning coffee) can be as impactful as a per-day framing at influencing product purchase intention. Either an implicit comparison via per-day framing, or an explicit petty cash comparison will result in significantly higher perceived values. In the field of sequence preference, Read and Powell (2002) labeled this type of thinking as “Ideal consumption,” as people tend to choose the sequence that they believe as appropriate (Chapman, 1996). Read and Powell (2002) also found a strong preference for constant sequences, mostly related to reasons of “convenience” or “the ease with which money can be managed.” In our study, the per-day reframed rising or falling profile can only be expressed as a rising or falling sequence of per-day loan costs, i.e., there are three different per-day loan costs in three years, making the petty cash comparison less obvious. Therefore, rising and falling profiles are perceived as more difficult to manage than constant profiles.

## Conclusion

Previous studies have shown a consistent preference for the falling sequence in loan repayment plans, suggesting that banks need to develop loan schemes in which the repayments are concentrated at the beginning of the loan term. However, our results show that consumers follow a comparison-based decision making process rather than optimization when evaluating temporally reframed loan offerings. Individuals preferred the falling over the constant profile only if the interest rate was 10% and the loan profiles were described in a per-year form. Otherwise, they preferred the constant profile. Therefore, regardless of the amply evidence supporting the improving sequence effect, borrowers may still prefer the level payment loans, especially when the loan profiles are expressed in a per-day form.

In general, we found that the improving sequence effect existed in a loan context and the DUM was violated. However, the violation of the DUM in the 0% interest condition cannot be explained by any discount function. Thus, we propose that future studies in sequence effect may also consider psychological reasons and comparison-based decision making process. However, there are limitations that need to be addressed in future studies. First, the study is limited in external validity in that respondents are not a representative sample from any particular population (all MBA students from the same university). Furthermore, the generalizability of the findings is limited in that the loan stimuli are entirely hypothetical based on a fictional job scenario provided to the students. Future research should design the experiment based on participants’ real-life job and financial backgrounds.

## Data Availability Statement

The datasets generated for this study are available on request to the corresponding author.

## Ethics Statement

Ethical review and approval was not required for the study on human participants in accordance with the local legislation and institutional requirements. The patients/participants provided their written informed consent to participate in this study.

## Author Contributions

YL designed research and wrote the manuscript. CL and HH collected data. JW and XZ analyzed data. All authors contributed to the article and approved the submitted version.

## Conflict of Interest

The authors declare that the research was conducted in the absence of any commercial or financial relationships that could be construed as a potential conflict of interest.
