# Supplementary material for: Price Attractiveness and Price Complexity: Why People Prefer Level-Payment Loans
Source: Front Psychol. 2021 Jun 10;12:532696. doi: 10.3389/fpsyg.2021.532696 (PMC8222506; doi:10.3389/fpsyg.2021.532696)
Supplement: Supplementary file 1 [file Data_Sheet_1.pdf]

## Appendix A

Table A1a Monthly Amortization plan of 120,000 car loan at 10% loan rate (falling)

| Month | Payment | Interest | Principal repaid | Outstanding principal |
|---|---|---|---|---|
| 0 | | | | ￥120,000.0 |
| 1 | ￥6,000.00 | ￥1,000.0 | ￥5,000.0 | ￥115,000.0 |
| 2 | ￥5,958.30 | ￥958.3 | ￥5,000.0 | ￥110,000.0 |
| 3 | ￥5,916.70 | ￥916.7 | ￥5,000.0 | ￥105,000.0 |
| 4 | ￥5,875.00 | ￥875.0 | ￥5,000.0 | ￥100,000.0 |
| 5 | ￥5,833.30 | ￥833.3 | ￥5,000.0 | ￥95,000.0 |
| 6 | ￥5,791.70 | ￥791.7 | ￥5,000.0 | ￥90,000.0 |
| 7 | ￥5,750.00 | ￥750.0 | ￥5,000.0 | ￥85,000.0 |
| 8 | ￥5,708.30 | ￥708.3 | ￥5,000.0 | ￥80,000.0 |
| 9 | ￥5,666.70 | ￥666.7 | ￥5,000.0 | ￥75,000.0 |
| 10 | ￥5,625.00 | ￥625.0 | ￥5,000.0 | ￥70,000.0 |
| 11 | ￥5,583.30 | ￥583.3 | ￥5,000.0 | ￥65,000.0 |
| 12 | ￥5,541.70 | ￥541.7 | ￥5,000.0 | ￥60,000.0 |
| 13 | ￥3,833.30 | ￥500.0 | ￥3,333.3 | ￥56,666.7 |
| 14 | ￥3,805.50 | ￥472.2 | ￥3,333.3 | ￥53,333.3 |
| 15 | ￥3,777.70 | ￥444.4 | ￥3,333.3 | ￥50,000.0 |
| 16 | ￥3,750.00 | ￥416.7 | ￥3,333.3 | ￥46,666.7 |
| 17 | ￥3,722.20 | ￥388.9 | ￥3,333.3 | ￥43,333.3 |
| 18 | ￥3,694.40 | ￥361.1 | ￥3,333.3 | ￥40,000.0 |
| 19 | ￥3,666.60 | ￥333.3 | ￥3,333.3 | ￥36,666.7 |
| 20 | ￥3,638.90 | ￥305.6 | ￥3,333.3 | ￥33,333.3 |
| 21 | ￥3,611.10 | ￥277.8 | ￥3,333.3 | ￥30,000.0 |
| 22 | ￥3,583.30 | ￥250.0 | ￥3,333.3 | ￥26,666.7 |
| 23 | ￥3,555.50 | ￥222.2 | ￥3,333.3 | ￥23,333.3 |
| 24 | ￥3,527.70 | ￥194.4 | ￥3,333.3 | ￥20,000.0 |
| 25 | ￥1,833.40 | ￥166.7 | ￥1,666.7 | ￥18,333.3 |
| 26 | ￥1,819.50 | ￥152.8 | ￥1,666.7 | ￥16,666.7 |
| 27 | ￥1,805.60 | ￥138.9 | ￥1,666.7 | ￥15,000.0 |
| 28 | ￥1,791.70 | ￥125.0 | ￥1,666.7 | ￥13,333.3 |
| 29 | ￥1,777.80 | ￥111.1 | ￥1,666.7 | ￥11,666.7 |
| 30 | ￥1,763.90 | ￥97.2 | ￥1,666.7 | ￥10,000.0 |
| 31 | ￥1,750.00 | ￥83.3 | ￥1,666.7 | ￥8,333.3 |
| 32 | ￥1,736.10 | ￥69.4 | ￥1,666.7 | ￥6,666.7 |
| 33 | ￥1,722.30 | ￥55.6 | ￥1,666.7 | ￥5,000.0 |
| 34 | ￥1,708.40 | ￥41.7 | ￥1,666.7 | ￥3,333.3 |
| 35 | ￥1,694.50 | ￥27.8 | ￥1,666.7 | ￥1,666.7 |
| 36 | ￥1,680.60 | ￥13.9 | ￥1,666.7 | ￥0.0 |

Table A1b Monthly Amortization plan of 120,000 car loan at 10% loan rate (constant)

| Month | Payment | Interest | Principal repaid | Outstanding principal |
|---|---|---|---|---|
| 0 | | | | ￥120,000.0 |
| 1 | ￥3,872.1 | ￥1,000.0 | ￥2,872.1 | ￥117,127.9 |
| 2 | ￥3,872.1 | ￥976.1 | ￥2,896.0 | ￥114,231.9 |
| 3 | ￥3,872.1 | ￥951.9 | ￥2,920.1 | ￥111,311.8 |
| 4 | ￥3,872.1 | ￥927.6 | ￥2,944.5 | ￥108,367.3 |
| 5 | ￥3,872.1 | ￥903.1 | ￥2,969.0 | ￥105,398.3 |
| 6 | ￥3,872.1 | ￥878.3 | ￥2,993.7 | ￥102,404.6 |
| 7 | ￥3,872.1 | ￥853.4 | ￥3,018.7 | ￥99,385.9 |
| 8 | ￥3,872.1 | ￥828.2 | ￥3,043.8 | ￥96,342.1 |
| 9 | ￥3,872.1 | ￥802.9 | ￥3,069.2 | ￥93,272.9 |
| 10 | ￥3,872.1 | ￥777.3 | ￥3,094.8 | ￥90,178.1 |
| 11 | ￥3,872.1 | ￥751.5 | ￥3,120.6 | ￥87,057.5 |
| 12 | ￥3,872.1 | ￥725.5 | ￥3,146.6 | ￥83,910.9 |
| 13 | ￥3,872.1 | ￥699.3 | ￥3,172.8 | ￥80,738.1 |
| 14 | ￥3,872.1 | ￥672.8 | ￥3,199.2 | ￥77,538.9 |
| 15 | ￥3,872.1 | ￥646.2 | ￥3,225.9 | ￥74,312.9 |
| 16 | ￥3,872.1 | ￥619.3 | ￥3,252.8 | ￥71,060.2 |
| 17 | ￥3,872.1 | ￥592.2 | ￥3,279.9 | ￥67,780.3 |
| 18 | ￥3,872.1 | ￥564.8 | ￥3,307.2 | ￥64,473.0 |
| 19 | ￥3,872.1 | ￥537.3 | ￥3,334.8 | ￥61,138.3 |
| 20 | ￥3,872.1 | ￥509.5 | ￥3,362.6 | ￥57,775.7 |
| 21 | ￥3,872.1 | ￥481.5 | ￥3,390.6 | ￥54,385.1 |
| 22 | ￥3,872.1 | ￥453.2 | ￥3,418.9 | ￥50,966.2 |
| 23 | ￥3,872.1 | ￥424.7 | ￥3,447.3 | ￥47,518.9 |
| 24 | ￥3,872.1 | ￥396.0 | ￥3,476.1 | ￥44,042.8 |
| 25 | ￥3,872.1 | ￥367.0 | ￥3,505.0 | ￥40,537.8 |
| 26 | ￥3,872.1 | ￥337.8 | ￥3,534.2 | ￥37,003.5 |
| 27 | ￥3,872.1 | ￥308.4 | ￥3,563.7 | ￥33,439.8 |
| 28 | ￥3,872.1 | ￥278.7 | ￥3,593.4 | ￥29,846.4 |
| 29 | ￥3,872.1 | ￥248.7 | ￥3,623.3 | ￥26,223.1 |
| 30 | ￥3,872.1 | ￥218.5 | ￥3,653.5 | ￥22,569.5 |
| 31 | ￥3,872.1 | ￥188.1 | ￥3,684.0 | ￥18,885.6 |
| 32 | ￥3,872.1 | ￥157.4 | ￥3,714.7 | ￥15,170.9 |
| 33 | ￥3,872.1 | ￥126.4 | ￥3,745.6 | ￥11,425.2 |
| 34 | ￥3,872.1 | ￥95.2 | ￥3,776.9 | ￥7,648.4 |
| 35 | ￥3,872.1 | ￥63.7 | ￥3,808.3 | ￥3,840.1 |
| 36 | ￥3,872.1 | ￥32.0 | ￥3,840.1 | ￥0.0 |

Table A1c Monthly Amortization plan of 120,000 car loan at 10% loan rate (rising)

| Month | Payment | Interest | Principal repaid | Outstanding principal |
|---|---|---|---|---|
| 0 | | | | ￥120,000.0 |
| 1 | ￥2,666.70 | ￥1,000.0 | ￥1,666.7 | ￥118,333.3 |
| 2 | ￥2,652.80 | ￥986.1 | ￥1,666.7 | ￥116,666.7 |
| 3 | ￥2,638.90 | ￥972.2 | ￥1,666.7 | ￥115,000.0 |
| 4 | ￥2,625.00 | ￥958.3 | ￥1,666.7 | ￥113,333.3 |
| 5 | ￥2,611.10 | ￥944.4 | ￥1,666.7 | ￥111,666.7 |
| 6 | ￥2,597.30 | ￥930.6 | ￥1,666.7 | ￥110,000.0 |
| 7 | ￥2,583.40 | ￥916.7 | ￥1,666.7 | ￥108,333.3 |
| 8 | ￥2,569.50 | ￥902.8 | ￥1,666.7 | ￥106,666.7 |
| 9 | ￥2,555.60 | ￥888.9 | ￥1,666.7 | ￥105,000.0 |
| 10 | ￥2,541.70 | ￥875.0 | ￥1,666.7 | ￥103,333.3 |
| 11 | ￥2,527.80 | ￥861.1 | ￥1,666.7 | ￥101,666.7 |
| 12 | ￥2,513.90 | ￥847.2 | ￥1,666.7 | ￥100,000.0 |
| 13 | ￥4,166.60 | ￥833.3 | ￥3,333.3 | ￥96,666.7 |
| 14 | ￥4,138.90 | ￥805.6 | ￥3,333.3 | ￥93,333.3 |
| 15 | ￥4,111.10 | ￥777.8 | ￥3,333.3 | ￥90,000.0 |
| 16 | ￥4,083.30 | ￥750.0 | ￥3,333.3 | ￥86,666.7 |
| 17 | ￥4,055.50 | ￥722.2 | ￥3,333.3 | ￥83,333.3 |
| 18 | ￥4,027.70 | ￥694.4 | ￥3,333.3 | ￥80,000.0 |
| 19 | ￥4,000.00 | ￥666.7 | ￥3,333.3 | ￥76,666.7 |
| 20 | ￥3,972.20 | ￥638.9 | ￥3,333.3 | ￥73,333.3 |
| 21 | ￥3,944.40 | ￥611.1 | ￥3,333.3 | ￥70,000.0 |
| 22 | ￥3,916.60 | ￥583.3 | ￥3,333.3 | ￥66,666.7 |
| 23 | ￥3,888.90 | ￥555.6 | ￥3,333.3 | ￥63,333.3 |
| 24 | ￥3,861.10 | ￥527.8 | ￥3,333.3 | ￥60,000.0 |
| 25 | ￥5,500.00 | ￥500.0 | ￥5,000.0 | ￥55,000.0 |
| 26 | ￥5,458.30 | ￥458.3 | ￥5,000.0 | ￥50,000.0 |
| 27 | ￥5,416.70 | ￥416.7 | ￥5,000.0 | ￥45,000.0 |
| 28 | ￥5,375.00 | ￥375.0 | ￥5,000.0 | ￥40,000.0 |
| 29 | ￥5,333.30 | ￥333.3 | ￥5,000.0 | ￥35,000.0 |
| 30 | ￥5,291.70 | ￥291.7 | ￥5,000.0 | ￥30,000.0 |
| 31 | ￥5,250.00 | ￥250.0 | ￥5,000.0 | ￥25,000.0 |
| 32 | ￥5,208.30 | ￥208.3 | ￥5,000.0 | ￥20,000.0 |
| 33 | ￥5,166.70 | ￥166.7 | ￥5,000.0 | ￥15,000.0 |
| 34 | ￥5,125.00 | ￥125.0 | ￥5,000.0 | ￥10,000.0 |
| 35 | ￥5,083.30 | ￥83.3 | ￥5,000.0 | ￥5,000.0 |
| 36 | ￥5,041.70 | ￥41.7 | ￥5,000.0 | ￥-0.0 |

Table A2a  Per-year and per-day reframed payments at 10% loan rate (falling)

| Year | Principal repaid | Interest | Per-year payment | Per-day payment |
|---|---|---|---|---|
| year 1 (month 1-12) | ￥60,000.0 | ￥9,250.0 | ￥69,250.0 | ￥189.7 |
| year 2 (month 13-24) | ￥40,000.0 | ￥4,166.7 | ￥44,166.7 | ￥121.0 |
| year 3 (month 25-26) | ￥20,000.0 | ￥1,083.3 | ￥21,083.3 | ￥57.8 |
| total | ￥120,000.0 | ￥14,500.0 | ￥134,500.0 | |

*Note.* Each year's value is the sum of 12 months values in that year. See tableA1a.

Table A2b  Per-year and per-day reframed payments at 10% loan rate (constant)

| Year | Principal repaid | Interest | Per-year payment | Per-day payment |
|---|---|---|---|---|
| year 1 (month 1-12) | ￥36,089.1 | ￥10,375.7 | ￥46,464.7 | ￥127.3 |
| year 2 (month 13-24) | ￥39,868.1 | ￥6,596.7 | ￥46,464.7 | ￥127.3 |
| year 3 (month 25-26) | ￥44,042.8 | ￥2,421.9 | ￥46,464.7 | ￥127.3 |
| total | ￥120,000.0 | ￥19,394.2 | ￥139,394.2 | |

*Note.* Each year's value is the sum of 12 months values in that year. See tableA1b.

Table A2c  Per-year and per-day reframed payments at 10% loan rate (rising)

| Year | Principal repaid | Interest | Per-year payment | Per-day payment |
|---|---|---|---|---|
| year 1 (month 1-12) | ￥20,000.0 | ￥11,083.3 | ￥31,083.3 | ￥85.2 |
| year 2 (month 13-24) | ￥40,000.0 | ￥8,166.7 | ￥48,166.7 | ￥132.0 |
| year 3 (month 25-26) | ￥60,000.0 | ￥3,250.0 | ￥63,250.0 | ￥173.3 |
| total | ￥120,000.0 | ￥22,500.0 | ￥142,500.0 | |

*Note.* Each year's value is the sum of 12 months values in that year. See tableA1c.
