# Supplementary material for: Price Attractiveness and Price Complexity: Why People Prefer Level-Payment Loans
Source: Front Psychol. 2021 Jun 10;12:532696. doi: 10.3389/fpsyg.2021.532696 (PMC8222506; doi:10.3389/fpsyg.2021.532696)
Supplement: Supplementary file 2 [file Data_Sheet_2.pdf]

## Appendix B

Questionnaires

These questionnaires must be anonymous and the information provided will not allow identifying, in any case, the respondent of each questionnaire. Please, respond to the questionnaires with total sincerity. It is preferable not to do them than to give insincere answers. We sincerely appreciate your participation and the time you dedicate to us. Thank you very much.

Age: ………………………………………………………………

Gender: Man …………………Woman ……………………

# INSTRUCTIONS

Imagine the following situation. You are working in a large company where you currently earn 10,000 Yuan per month after taxes, and your job is guaranteed for at least the next 3 years. You want to buy a new car from a car dealer. The price of the car is 120,000 Yuan. However, currently you do not have enough money to buy it so the dealer offers you a loan for this exact amount. This loan is for 3 years.You may select from the following 3 payment proposals.

Your tasks:

1)Pleasetry to score EACH PROPOSAL from 1 to 7, where 1 is the score to be assigned to the proposal least favorable and 7 to the most favorable proposal.

2)Please try to evaluate price complexity of EACH PROPOSAL, where 1 is“ not at all complex”, and 7 is “extremely complex”.

3)Please try to evaluate price attractiveness of EACH PROPOSAL, where 1 is“not at all attractive” and 7 is “extremely attractive”.

Questionnaire Group 1(10% car loan, per-year reframed)

<table>
<tr>
<td>PROPOSAL #1 (falling)<br>The monthly principal repayments of this loan are ¥5,000 in year 1, ¥3,333.3 in year 2, and ¥1,666.7 in year 3. Each month you will pay 10%p.a. (per annum) interest for the outstanding debt.</td>
<td>PROPOSAL #2(constant)<br>This plan is a fully amortized level-payment car loan. The monthly payments are identical over the life of loan. Each monthly payment includes principal repayment and 10% p.a. interest for the outstanding debt.</td>
<td>PROPOSAL #3(rising)<br>The monthly principal repayments of this loan are¥1,666.7 in year 1, ¥3,333.3 in year 2, and ¥5,000 in year 3. Each month you will pay 10% p.a. interest for the outstanding debt.</td>
</tr>
<tr>
<td>Per-year payments
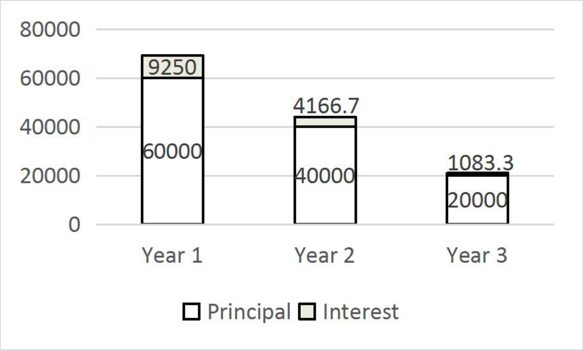

Only ¥69,250 in year 1, ¥44,166.7 in year 2, and ¥21,083.3 in year 3. Total payment is ¥134,500.</td>
<td>Per-year payments
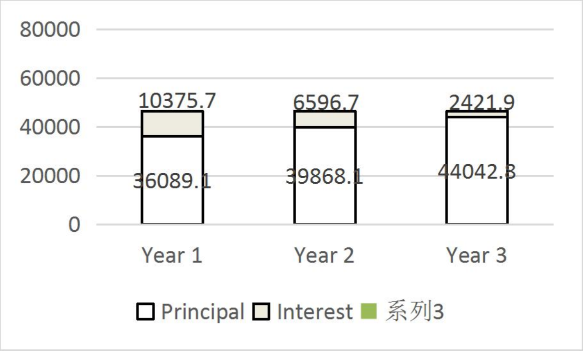

Only ¥46,464.75 per year.<br>Total payment is ¥139,394.25</td>
<td>Per-year payments
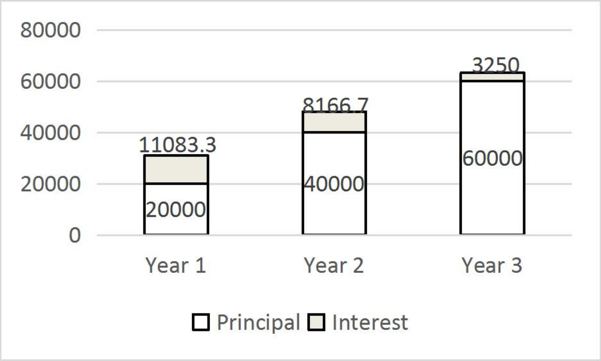

Only ¥31,083.3 in year 1, ¥48,166.7 in year 2, and ¥63,250 in year 3. Total payment is ¥142,500.</td>
</tr>
<tr>
<td>Score PROPOSAL #1 (1 ~ 7)</td>
<td>Score PROPOSAL #2 (1 ~ 7)</td>
<td>Score PROPOSAL #3 (1 ~ 7)</td>
</tr>
<tr>
<td>Price complexity #1 (1 ~ 7)</td>
<td>Price complexity #2 (1 ~ 7)</td>
<td>Price complexity #3 (1 ~ 7)</td>
</tr>
<tr>
<td>Price attractiveness #1 (1 ~ 7)</td>
<td>Price attractiveness #2 (1 ~ 7)</td>
<td>Price attractiveness #3 (1 ~ 7)</td>
</tr>
</table>

Questionnaire Group 2 (10% car loan, per-day reframed)

| | | |
|---|---|---|
| PROPOSAL #1 (falling)<br>The monthly principal repayments of this loan are ¥5,000 in year 1, ¥3,333.3 in year 2, and ¥1,666.7 in year 3. Each month you will pay 10%p.a. (per annum) interest for the outstanding debt. | PROPOSAL #2(constant)<br>This plan is a fully amortized level-payment car loan. The monthly payments are identical over the life of loan. Each monthly payment includes principal repayment and 10% p.a. interest for the outstanding debt. | PROPOSAL #3(rising)<br>The monthly principal repayments of this loan are¥1,666.7 in year 1, ¥3,333.3 in year 2, and ¥5,000 in year 3. Each month you will pay 10% p.a. interest for the outstanding debt. |
| Per-day payments<br>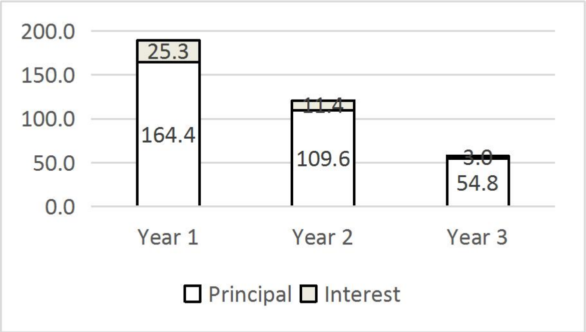<br>Only ¥189.7 per day in year 1, ¥121 per day in year 2, and ¥57.8 per day in year 3.<br>Total payment is ¥134,500. | Per-day payments<br>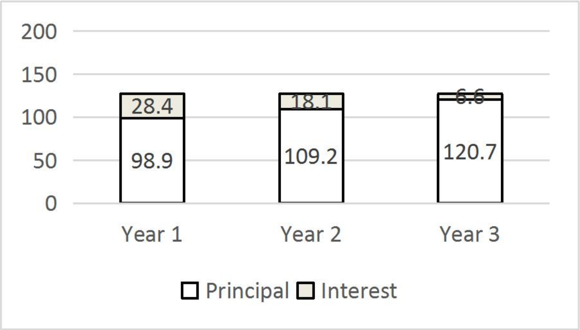<br>Only ¥127.3 per day.<br>Total payment is ¥139,394.25 | Per-day payments<br>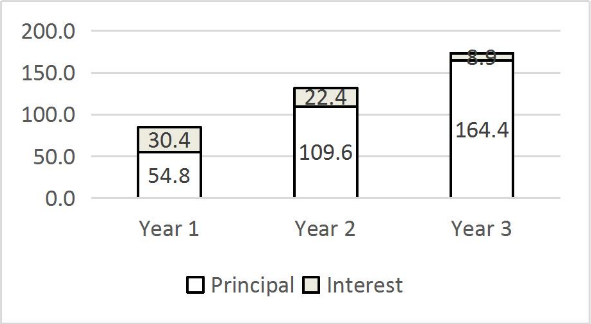<br>Only ¥85.2 per day in year1, ¥132 per day in year 2, and ¥173.3 per day in year 3.<br>Total payment is ¥142,500. |
| Score PROPOSAL #1 (1 ~ 7) | Score PROPOSAL #2 (1 ~ 7) | Score PROPOSAL #3 (1 ~ 7) |
| Price complexity #1 (1 ~ 7) | Price complexity #2 (1 ~ 7) | Price complexity #3 (1 ~ 7) |
| Price attractiveness #1 (1 ~ 7) | Price attractiveness #2 (1 ~ 7) | Price attractiveness #3 (1 ~ 7) |

Questionnaire Group 3 (0% car loan, per-year reframed)

| PROPOSAL #1 (falling)<br>The monthly principal repayments of this loan are ¥5,000 in year 1, ¥3,333.3 in year 2, and ¥1,666.7 in year 3. No interest is charged. | PROPOSAL #2(constant)<br>This plan is a fully amortized level-payment car loan. The monthly payments are identical over the life of loan. No interest is charged. | PROPOSAL #3(rising)<br>The monthly principal repayments of this loan are¥1,666.7 in year 1, ¥3,333.3 in year 2, and ¥5,000 in year 3. No interest is charged. |
|---|---|---|
| Per-year payments<br>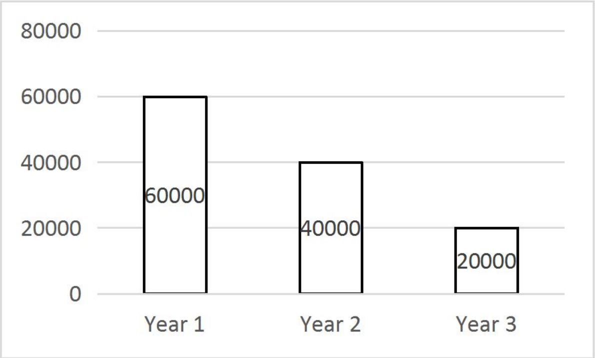<br>Only ¥60,000 in year 1, ¥40,000 in year 2, and ¥20,000 in year 3.<br>Total payment is ¥120,000. | Per-year payments<br>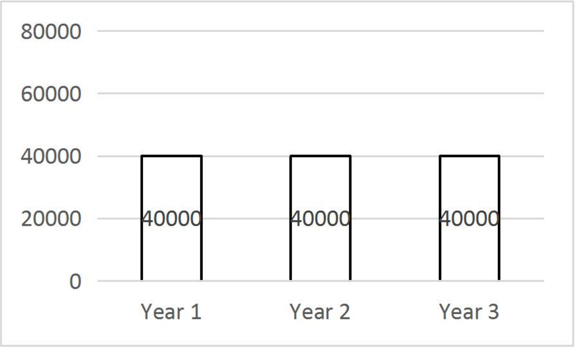<br>Only ¥40,000 per year.<br>Total payment is ¥120,000. | Per-year payments<br>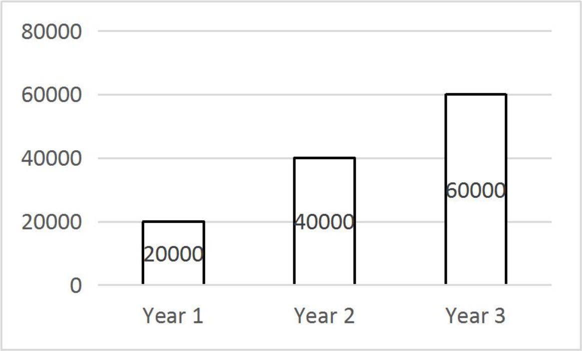<br>Only ¥20,000 in year 1, ¥40,000 in year 2, and ¥60,000 in year 3.<br>Total payment is ¥120,000. |
| Score PROPOSAL #1 (1 ~ 7) | Score PROPOSAL #2 (1 ~ 7) | Score PROPOSAL #3 (1 ~ 7) |
| Price complexity #1 (1 ~ 7) | Price complexity #2 (1 ~ 7) | Price complexity #3 (1 ~ 7) |
| Price attractiveness #1 (1 ~ 7) | Price attractiveness #2 (1 ~ 7) | Price attractiveness #3 (1 ~ 7) |

Questionnaire Group 4 (0% car loan, per-day reframed)

| PROPOSAL #1 (falling) | PROPOSAL #2(constant) | PROPOSAL #3(rising) |
|---|---|---|
| The monthly principal repayments of this loan are ¥5,000 in year 1, ¥3,333.3 in year 2, and ¥1,666.7 in year 3. No interest is charged. | This plan is a fully amortized level-payment car loan. The monthly payments are identical over the life of loan. No interest is charged. | The monthly principal repayments of this loan are¥1,666.7 in year 1, ¥3,333.3 in year 2, and ¥5,000 in year 3. No interest is charged. |
| Per-day payments<br>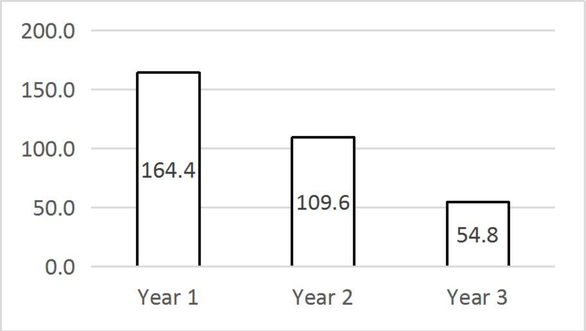<br>Only ¥164.4 per day in year 1, ¥109.6 per day in year 2, and ¥54.8 per day in year 3.<br>Total payment is ¥120,000. | Per-day payments<br>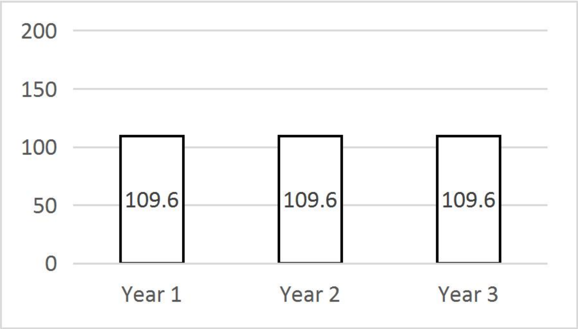<br>Only ¥109.6 per day.<br>Total payment is ¥120,000. | Per-day payments<br>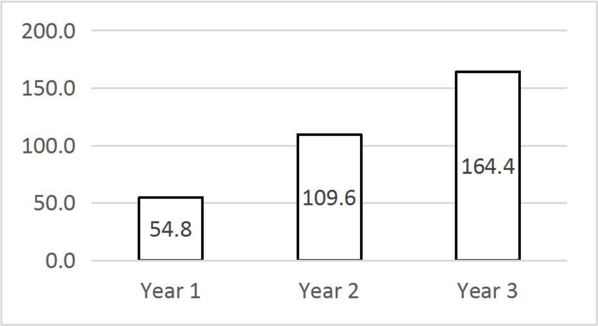<br>Only ¥54.8 per day in year 1, ¥109.6 per day in year 2, and ¥164.4 per day in year 3.<br>Total payment is ¥120,000. |
| Score PROPOSAL #1 (1 ~ 7) | Score PROPOSAL #2 (1 ~ 7) | Score PROPOSAL #3 (1 ~ 7) |
| Price complexity #1 (1 ~ 7) | Price complexity #2 (1 ~ 7) | Price complexity #3 (1 ~ 7) |
| Price attractiveness #1 (1 ~ 7) | Price attractiveness #2 (1 ~ 7) | Price attractiveness #3 (1 ~ 7) |
